# Evaluation of Chlamydia Partner Notification Practices and Use of the “Let Them Know” Website by Family Planning Clinicians in Australia: Cross-Sectional Study

**DOI:** 10.2196/jmir.5441

**Published:** 2016-06-24

**Authors:** Rebecca J Guy, Joanne M Micallef, Julie Mooney-Somers, Muhammad S Jamil, Caroline Harvey, Deborah Bateson, Caroline van Gemert, Handan Wand, John Kaldor

**Affiliations:** ^1^ The Kirby Institute, UNSW Australia Sydney Australia; ^2^ Centre for Values, Ethics and the Law in Medicine, University of Sydney Sydney Australia; ^3^ Institute for Urban Indigenous Health Brisbane Australia; ^4^ Family Planning New South Wales Sydney Australia; ^5^ Discipline of Obstetrics, Gynaecology and Neonatology, The University of Sydney Sydney Australia; ^6^ Centre for Population Health, Burnet Institute Melbourne Australia

**Keywords:** Chlamydia trachomatis, partner notification, Internet

## Abstract

**Background:**

Chlamydia, caused by *Chlamydia trachomatis*, is the most common reportable infection in many developed countries. Testing, treatment, and partner notification (PN) are key strategies for chlamydia control. In 2008 the Let Them Know (LTK) PN website was established, which provided means for people to send anonymous PN messages by text messaging (short message service, SMS), email, or letter.

**Objective:**

We evaluated PN practices among Australian family planning clinicians following chlamydia diagnosis and assessed how often clinicians refer their patients to the LTK website.

**Methods:**

A mixed methods approach included a Web-based cross-sectional survey of Australian family planning clinicians to examine PN attitudes and practices and focus groups to explore the context of LTK website use.

**Results:**

Between May 2012 and June 2012, all clinicians from 29 different family planning services (n=212) were invited to complete the survey, and 164 participated (response rate=77.4%); of the clinicians, 96.3% (158/164) were females, 56.1% (92/164) nurses, and 43.9% (72/164) doctors. More than half (62.2%, 92/148) agreed that PN was primarily the client's responsibility; however, 93.2% (138/148) agreed it was the clinician's responsibility to support the client in informing their partners by providing information or access to resources. Almost half (49.4%, 76/154) of the clinicians said that they always or usually referred clients to the LTK website, with variation across clinics in Australian states and territories (0%-77%). Eleven focus groups among 70 clinicians at 11 family planning services found that the LTK website had been integrated into routine practice; that it was particularly useful for clients who found it difficult to contact partners; and that the LTK letters and fact sheets were useful. However, many clinicians were not aware of the website and noted a lack of internal clinic training about LTK.

**Conclusions:**

The LTK website has become an important PN tool for family planning clinicians. The variation in referral of patients to the LTK website and lack of awareness among some clinicians suggest further promotion of the website, PN training, and clinic protocols are warranted.

## Introduction

Chlamydia, caused by *Chlamydia trachomatis*, is the most common reportable infection in the United States, Australia, and European countries [1-3]. In 2014 more than 1.4 million new diagnoses were reported in the United States [1], and 86,000 chlamydia cases were notified in Australia [3]. However, more than three-quarters (76%) of infections remain undiagnosed at any point in time [4]. Testing, treatment, and partner notification (PN) are key strategies for chlamydia control. Partner notification and testing has been shown to reduce reinfection rates in index cases [5]. Mathematical modeling suggests that, in a population-wide screening program, the treatment of current partner is the most effective strategy for preventing reinfection of index cases and reducing further chlamydia transmission at the population level [6].

Clinical guidelines in the United Kingdom and Australia recommend testing and treating all sexual partners in the last 6 months [7,8], whereas US guidelines recommend to treat all partners in the last 2 months or the most recent partner if the last sexual contact was more than 2 months ago [9]. Despite clinicians recognizing its importance, PN has long been recognized as a challenge for clinicians and patients alike, because of the sensitivities involved in disclosing and informing [10]. Most clinicians report they would like additional supportive resources, including websites [10]. New, accessible, and convenient approaches are needed to inform partners of their potential disease exposure.

**Figure 1:**
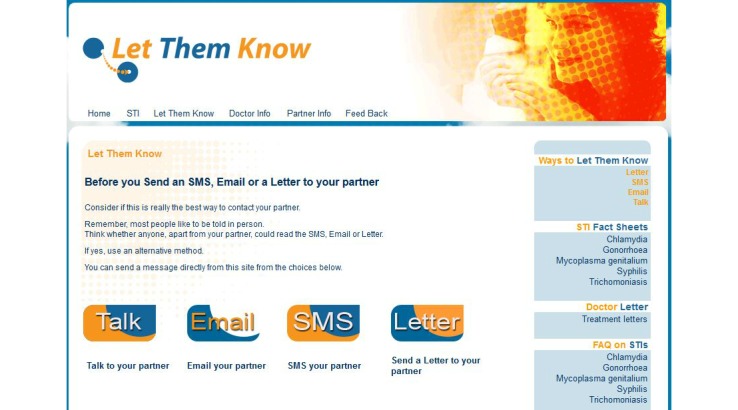
Let Them Know website interface screenshot.

In December 2008 the Let Them Know (LTK) website was launched in Australia and provided means for people to send anonymous PN messages by short message service (SMS) text messaging, email, or letter (Figure 1) [11]. The LTK website was the first in the world to enable young people to notify their partners anonymously using SMS text messaging, whereas other systems only offered electronic postcards or email. The LTK website also includes fact sheets for sexually transmitted infections (STIs) and letter templates with testing and treatment recommendations for the partners to pass on to their doctors. The website was developed by the Melbourne Sexual Health Centre in Victoria with some information on the website (contact details and letters) specific to this clinic; it was later adapted for use in New South Wales and then Australia-wide by customization to local resources [11]. The website was developed originally for chlamydia and later adapted for other STIs [12]. No specific promotion of the website occurred across Australia.

In the past few years, other Internet-based PN services have been developed [13-18], and websites have also been used to promote chlamydia screening in young people and providers [19,20]. The “WhyTest” service for gay men in Australia included SMS text messaging and email notification [13]; the “inSPOT” service in the United States enabled notification by postcards and emails [16] and has since been replicated elsewhere [17,18]; and in the Netherlands, the “suggest a test” service offered SMS text messaging, email, postal letter, or a personal message to notify sexual contacts [14]. Evaluation of these PN websites has mainly focused on website usage and showed the SMS text messaging function is far more popular than email [13,14].

Because of the inherent nature of these services, demonstration of effectiveness is very challenging. To our knowledge only one randomized controlled trial has evaluated the impact of PN websites (inSPOT) on partner treatment among men who have sex with men [15]; however, the website only provided email and postcard services, which have been shown to be less popular than SMS text messaging. A previous evaluation of the LTK website has shown that people who used the service reported they were more likely to contact a partner because of the website [12], which should ultimately lead to a greater uptake of PN overall. However, studies in the United States show that awareness and uptake among the target group is low [17,18]. It is possible that the low uptake is due to clinicians not promoting the services actively to their clients.

We evaluated PN practices among family planning clinicians following chlamydia diagnosis and assessed how often clinicians refer patients to the LTK website to notify their partners. Although the LTK website offered PN for other STIs, we focused on chlamydia as the prevalence in young people in Australia is higher compared with other STIs including gonorrhea [3]. The study was conducted in the context of a broader study assessing chlamydia testing and management practices at Australian family planning clinics.

## Methods

### Setting

The study was undertaken among clinicians, both doctors and nurses, working in Australian family planning clinics, which provide sexual and reproductive health (SRH) services and are located in all Australian states and territories. These clinics have a high caseload of young people aged 16-29 years who are sexually active and at risk of chlamydia infection, with more than 90% females [21]. The clinics are run by independent, nongovernment, not-for-profit organizations responsible to a voluntary board of directors.

Nurses in family planning clinics work in various roles within and between states. Most nurses have SRH qualifications but the scope of practice includes specialized SRH advanced practice nurses, which may include limited medication supply, various autonomous clinical consulting roles, and phone advice or clinical practice support. State or territory legislation governs nurse medication supply, which affects the degree of autonomy in treating chlamydia. For example, in some states most family planning nurses are authorized under legislated drug therapy protocols to autonomously supply treatment for people diagnosed with chlamydia and their contacts and would, as part of their duty of care, provide information on and support for PN. Whereas nurses in other jurisdictions may need to refer all chlamydia cases to family planning doctors for management and would generally not be involved in PN or support of clients.

### Study Design

We used a mixed methods design involving a cross-sectional survey to examine chlamydia testing and management, and PN attitudes and practices among clinicians, and focus groups to explore the context of chlamydia management and PN.

#### Cross-Sectional Survey

All doctors and nurses recorded as being employed clinically at all 29 Australian family planning clinics (as of April 2012) were invited to complete a survey by email. Researchers sent an introductory email to the clinic manager or administrative officer, who circulated it among clinic staff members. The email contained the link to the Web-based survey. The clinic manager also provided the number of individual staff working at each clinic to calculate the response rate. Posters were also displayed in staff areas to raise awareness of the survey. After 4 weeks a reminder was sent to the clinic representative to encourage nonresponders to complete the survey.

The Web-based survey captured clinician demographics, experience, attitudes, and practices related to chlamydia testing and management (retesting and PN). Most questions sought responses on a 5-point Likert scale from strongly agree to strongly disagree or a 4-point scale of always, usually, sometimes, or never. The questionnaire was tested with clinicians for its content, language, and feasibility of questionnaire length.

Descriptive statistics were used to examine the responses to survey questions about chlamydia PN attitudes and practices.

#### Focus Groups

The cross-sectional survey and focus groups were conducted sequentially, not in parallel. We conducted surveys before the focus groups to allow us to use survey findings to purposively sample clinics [22]. To ensure a diverse sample of clinics we selected across a range of urban and regional clinics across Australia, client demographics (eg, youth focused), reported PN strategies, reported retesting strategies, and, finally, reported responses to screening in an asymptomatic client scenario. In selected clinics, the clinic representative invited all doctors and nurses to attend. Some clinics also invited health promotion officers. Focus groups were conducted over lunchtime or directly after clinic hours. Each group was facilitated by 2 researchers and audio recorded.

Focus groups began with a general discussion about the clinical setting, client population, and the local ethos around chlamydia prevention. Then in relation to (1) chlamydia testing, (2) PN and treatment (including the use of technology such as LTK), (3) retesting, they were asked to discuss an example of practice where things went well, how they know when things are working well, and how they think things could work well more often. Focus groups were transcribed and data analyzed using thematic analysis [23]. NVivo 10.0 was used to support analysis.

### Ethical Approval

Ethical approval was obtained from Family Planning NSW Ethics Committee and Family Planning Victoria Human Research Ethics Committee.

## Results

### Participants

Between May 2012 and June 2012, all clinicians employed by Australian family planning clinics (n=212) were invited to complete the Web-based survey and 164 participated, giving a response rate of 77.4%; of the clinicians, 96.3% (158/164) were females, 56.1% (92/164) were nurses, and 43.9% (72/164) were doctors. All 29 Australian family planning clinics were represented in the survey. More than half (56.1%, 92/164) of the participants were aged 45 years or older, 47.0% (77/164) had worked at the current organization for 6 years or more, 69.5% (114/164) as a clinician with a special interest in reproductive and sexual health, and 42.7% (70/164) worked at a family planning clinic less than 10 hours per week. About a third of the clinicians (31.7%, 52/164) managed more than 3 female clients with a positive chlamydia test result per month, 45.7% (75/164) saw 1-3 per month, and 20.1% (33/164) less than 1 per month (Table 1). Focus groups were held in 11 metropolitan and regional clinics across Australia and involved a total of 70 nurses, doctors, or health promotion officers (range 4-11 participants per clinic).

### Partner Notification Attitudes

**Figure 2:**
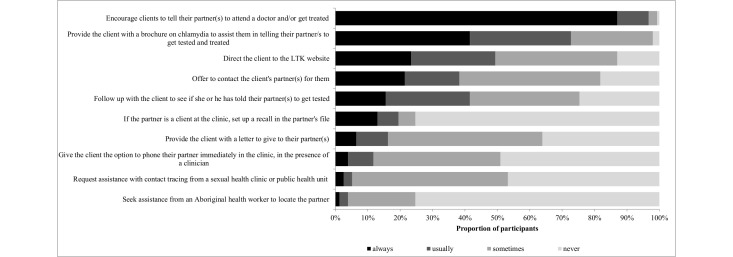
Partner notification attitudes among Australian family planning clinicians (n=148).

Almost all clinicians (99.3%, 147/148) strongly agreed or agreed that PN is an important strategy for preventing reinfection, and 97.3% (144/148) strongly agreed or agreed it is an important public health strategy to reduce the community prevalence of chlamydia. More than half (66.2%, 98/148) of the clinicians strongly agreed or agreed that PN is difficult as clients do not always feel comfortable talking to partners about chlamydia, and 47.3% (70/148) found it difficult because clients do not like to name their partners or are unable to name them (eg, they did not know the partner’s name or contact details). On the other hand, relatively few (12.2%, 18/148) strongly agreed or agreed that PN is too difficult to implement (Figure 2).

More than half (62.2%, 92/148) of the participants strongly agreed or agreed that PN was primarily the client's responsibility, whereas 10.1% (15/148) strongly agreed or agreed it was primarily the clinician's responsibility to notify the partner. Nevertheless, the vast majority (93.2%, 138/148) strongly agreed or agreed it was the clinician's responsibility to support the client in informing their partners by providing information or access to resources. Focus group strongly reflected these findings; for example:

We teach that you’re responsible for making sure they’re aware that their partners should be contacted and treated, and that the clinician is happy to help that process. Whether that’s through the Let Them Know website, or whether they actually want the clinician to do it themselves, which would be pretty unusual, I think. But the responsibility is to make sure the patient’s aware that the partner should be notified and treated, and tested. So there’s no - I don’t think any of the clinicians probably feel that it’s their responsibility to contact the partners.

**Table 1:** Description of Australian family planning clinician survey participants.

Characteristics	Sub-category	n (%)
Provider type	Nurse	92 (56.1)
	Doctor	72 (43.9)
Sex	Female	158 (96.3)
	Male	3 (1.8)
	Missing	3 (1.8)
Age, years	<35	26 (15.9)
	35-44	45 (27.4)
	45-54	58 (35.4)
	55+	34 (20.7)
	Missing	1 (0.6)
Clinic location	Urban	103 (62.8)
	Regional/remote	61 (37.2)
Years worked at current organization	<1	22 (13.4)
	1-3	32 (19.5)
	>3-5	31 (18.9)
	>5	77 (47.0)
	Missing	2 (1.2)
Years worked as a clinician with a special interest in sexual/reproductive health	<3	25 (15.2)
	3-5	24 (14.6)
	>5	114 (69.5)
	Missing	1 (0.6)
Hours per week providing clinical services in a general family planning service	<10	70 (42.7)
	10-20	61 (37.2)
	21-30	29 (17.7)
	≥31	3 (1.8)
	Missing	1 (0.6)
Approximate chlamydia test requested per week	None	2 (1.2)
	1-5	80 (48.8)
	6-10	50 (30.5)
	11-20	25 (15.2)
	>20	5 (3.0)
	Missing	2 (1.2)
Female clients with a positive chlamydia test managed	None	11 (6.7)
	<1 per month	22 (13.4)
	1-3 per month	75 (45.7)
	1-2 per week	36 (22.0)
	3-5 per week	14 (8.5)
	≥6 per week	2 (1.2)
	Missing	4 (2.4)
Male clients with a positive chlamydia test managed	None	34 (20.7)
	<1 per month	70 (42.7)
	1-3 per month 43 (26.2)	
	1-2 per week	12 (7.3)
	3-5 per week	2 (1.2)
	≥6 per week	0
	Missing	3 (1.8)

### Partner Notification Practices

**Figure 3:**
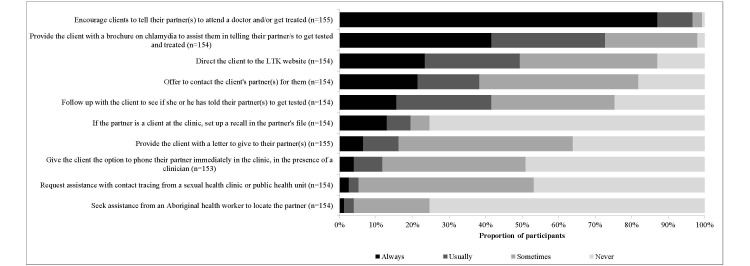
Partner notification practices among Australian family planning clinicians.

The most frequently reported strategy for PN was always or usually encouraging clients to undertake responsibility for communicating with sexual partners (96.8%, 150/155), followed by providing the clients with a brochure about chlamydia (72.7% 112/154), directing the clients to the LTK website (49.4%, 76/154), monitoring the clients to confirm they had notified their partner (41.6%, 64/154), and offering to contact the client’s partners (38.3%, 59/154; Figure 3). The majority of clinicians stated, consistent with Australian clinical guidelines [7,24], that they encouraged contacting all sexual partners in the past 6 months before the diagnosis (77.1%, 118/153), with 11.1% (14/153) stating 3 months or less, and 11.8% (18/153) stating 1 year or more. Focus groups revealed family planning clinics did not have specific protocols around the practice of PN; instead, individual clinicians tailored their approach to client need:

We have a policy around contact tracing, that we do it, but how it’s done depends on that individual client, really. It’s basically based on whether they can notify their partners. And if they can’t or they don’t want to, then we will do that but we don’t actually have a whole protocol for notifying patients and partners and stuff, do we?

### Partner Notification Barriers

Three key challenges to PN emerged in the focus groups. First, across focus groups there was uncertainty about what was expected of clinicians in relation to PN. As indicated in the survey findings, there was consensus on the importance of telling clients they should inform their contacts. However, clinicians were unsure how much effort they were expected to make:

Clinician: *However, I do find it quite difficult when you have this list of phone numbers and you don’t get a hold of someone, you know? And, like, how long do you keep on trying?*

Clinician: *You don’t even know if the number’s still current.*

Clinician: *Yes.*

Clinician: *That’s right.*

Clinician: *Yes. That’s where we need a policy.*

Second, some clinicians suggested it was hard to be enthusiastic about PN for chlamydia because it was not as serious a disease as gonorrhea, syphilis, or human immunodeficiency virus infection—“they’re kind of high-end STIs.” With these diseases clinicians described extensive PN:

If you had something, you know, that was more serious, for want of a better word ... we would then go all out to really make sure that person contacted their contacts ... but I think with chlamydia, because it's a bit familiar and common ... if the young person says yeah, she'll contact so-and-so, we just leave it at that a lot of the time.

Finally, clinicians in focus groups expressed a low level of confidence in their ability to tell if their PN efforts were successful. They rarely knew if clients had notified partners, and if they had, if those partners had sought treatment:

Yes, because we don’t know whether those contacts are going to their GP and getting tested or treated and unless we – unless everyone brings that contact in to us, we don’t really know the outcome.

Although some clinicians described formally following up with returning clients, most had to rely on trusting that clients were disclosing contacts to the clinician and that they would inform their contacts. Some did not feel confident that this trust was well placed:

Although sometimes I, kind of, get a sense that, “Oh, yes, you know, I’ll tell everybody,” and you just think, I don’t really know if you are going to do that.

### Partner Notification Facilitators

In focus groups, clinicians identified two key PN facilitators. First, many clinicians said that when they first discuss screening, they also prepare clients for the possibility of having to inform a contact of a positive result:

And making sure they’re aware of that, that we have to contact trace, before they have the test. That can be useful if they’re already prepared for that, if they get a positive test.

Clinicians described various strategies for explaining the importance of PN, including telling clients it was their legal or moral responsibility. Explaining the logic of PN was especially important where a client’s relationship with the sexual contact may be acrimonious:

And if you do not treat him, then he might give to other women, other women may give to other men and other men may end up give you - give back to you in the end.

The second facilitator was the low level of stigma associated with chlamydia among younger clients. This meant they were less embarrassed about having to inform a sexual contact and contacts were likely receptive to the information:

This reflects on the education that’s out there for young people. When I did talk about contact tracing to one girl, she just gave me the mobile numbers of all the men she’d had sex with in the last six months ... when I did phone each of these people, none of them were surprised or shocked or disbelieving about it. They said, “Yes, okay. Well, I’ll go to my doctor.” I was very surprised at how accepting the contacts were about this communication from me.

Providing easy access to screening for partners, clinics also let clients and partners know how easy and noninvasive specimen collection was:

Rather than thinking they’re going to have something stuck in their urethra or - I say to them, even the GP might not even need to actually even examine your partner if he doesn’t have symptoms. He might just have to really just wee in the jar and send it off for a test. It’s that easy.”

### Directing the Client to the Let Them Know Website

Of the participating clinicians, 23.4% (36/154) reported they always direct their clients to the LTK website, 26.0% (40/154) usually, 37.7% (58/154) sometimes, and 13.0% (20/154) never. According to profession, 49% of both doctors and nurses reported they always or usually directed their clients to LTK website. Always or usually directing the client to the LTK website varied by state or territory: 77.5% (31/40), 66.7% (2/3), 57.1% (8/14), 53.9% (7/13), 47.6% (10/21), 35.0% (7/20), 32.4% (11/34), and 0% (0/9). Also within states, where there were a number of family planning clinics, there was variation. In the state or territory where 0% always or usually directed clients to the website, two thirds of clinicians (66.7%) reported never directing clients to the website and others reported sometimes.

It was clear that while some clinicians promoted the use of the LTK website when they delivered training, awareness among family planning clinicians was not consistent. Some had never heard of it, others were unsure how it worked:

I need to go and look up, Let Them Know ...'Cause I haven’t seen or heard of those before. So I should go and do that …Leave that with us and we'll have a look.”

### Integration of the Let Them Know Website Into Routine Practice

The focus groups revealed the ways in which the LTK website was integrated into routine practice. For example, it could be raised during the phone call when a patient was being informed by the clinician of their positive chlamydia result:

I mean I usually tell them about that they need to notify their partners, and just check if they’re happy to do it or not...but they’re usually like, “Who knows where they are?” So I usually say there is a website, “Do you want it now or do you want to talk when you come in for your treatment?”

Clinicians also described introducing the LTK website during a face-to-face consultation, when it could be demonstrated to the client or used on the spot:

Clinician: *I often get them to do the Let Them Know site right there at the time because it’s easy to just pull it up while they’re screened.*

Clinician: *Yeah. While they’re there. Yeah.*

Clinician: *By the time you’ve pulled it up and said, “Well, this is how you do it and there it is.” It’s like, “come on, give me the number.”*

Clinician: *I say, “Have you got his number?” And they’ve got their phone there always.*

Clinician: *Let’s do it.*

Clinician: *They’ve got the number in their phone. You just do it.*

In focus groups, clinicians who did use the LTK website reported it was especially useful for patients who found it difficult to contact partners (these patients may previously have asked clinicians to make direct contact on their behalf):

I think that service [LTK] was good because I remember a couple of years back I think on two or three occasions I phoned a partner at the client’s request because they had a name and a phone number but didn’t want to do it themselves. And in those cases, now, they’ll use the Let Them Know website. But I agree, the majority of people say, “No, I’m going to tell them. I want to talk to them,” you know?

Among survey respondents who never directed their client to the LTK website, 90% said they would like access to this resource.

### Let Them Know Letters and Fact Sheets

Clinicians liked LTK for the letters and fact sheets;

I think that strategy of the letters that are accessible on the web are very useful for GPs to know about, to give the positive patient to give to their partner. And I think those. You know the letters that say - that they give to their boyfriend, then the boyfriend can take it to the GP. He doesn’t even have to say anything. And the letter says, “This patient’s partner has been diagnosed with chlamydia.”And it actually tells the GP what to treat with them as well. It says, “We suggest that you treat with, and test, and then based on the results, do further contact tracing.” And there’s an information sheet for the patients as well about what chlamydia is. But I like that actual letter for the GP.

## Discussion

To our knowledge this is the first study to evaluate PN practices and the use of the LTK website by family planning clinicians in Australia. We demonstrated that most clinicians take responsibility for supporting their clients to inform their sexual partners and the LTK website was widely used to achieve this goal. Almost half of the clinicians always or usually referred clients to the website, but with considerable variation across clinics in Australian states and territories. The LTK website was considered a useful tool, particularly when clients do not feel comfortable talking to partners about chlamydia.

We found that although family planning clinicians believed PN was primarily the client's responsibility, nearly all supported clients to inform their partners, thus understanding barriers to PN is important. Our survey showed that more than half of the family planning clinicians reported they found PN generally difficult as clients do not always feel comfortable talking to partners about chlamydia and often do not like to name their partners. Other barriers included uncertainty about what was expected of clinicians in relation to PN and doubt about the importance of PN for chlamydia. Facilitators of PN included preparing the client for a positive result when tested, letting clients and partners know how easy specimen collection was, and low levels of stigma about chlamydia in the community. The finding that clinicians were uncertain about what was expected is consistent with surveys of general practitioners in Australia where 45% (105/232) of clinicians were unsure how best to assist their patients with PN with considerable variation in the way PN was undertaken [10].

Despite these barriers, the LTK website was widely used for PN among family planning clinicians, and focus groups revealed that the LTK website was especially useful for patients who found it difficult to contact partners, patients who may previously have relied on clinicians making contact on their behalf. However, there was variation in LTK website use across different states and territories, which may reflect in part the place where it was developed and its subsequent adaption. The website was adapted first in Victoria and New South Wales, with clinics in both states having high proportions of clinicians who reported using LTK always or usually. However, there were clinics in other states where the website was adapted for use in later years, which also had higher proportion of clinicians using LTK, suggesting that the place of development may have played a role in greater use among clinicians, but that other factors also contributed. The LTK website was not formally promoted across Australia; rather, clinics that were aware of it integrated it into clinical practice. Also, the focus groups suggested variation in the formality of PN guidelines and PN training updates.

Despite clinicians recommending clients to use the LTK website, it may not necessarily translate to clients using the website or their partners seeking assessment and treatment. It was raised as a key barrier to PN generally, in that clinicians were unable to judge how successful the activity was. For example, evaluation of the “suggest a test” website in 2 cities in the Netherlands demonstrated that, of those intending to use the website, 23% notified partners using suggest a test and 20% of partners notified by suggest a test subsequently consulted a sexual health clinic [14]. To overcome this, some clinicians mentioned they often asked the client to use the LTK website during the follow-up treatment consultation, providing reassurance to the clinicians that PN had occurred. These findings have implications for general practice, where clinicians have reported not knowing how best to support patients with PN and time would generally be more limited than family planning clinics [10].

The uptake of the PN website among family planning clinicians is far greater than in Australian general practice. A recent study showed only 26% of Australian general practitioners always or usually directed clients diagnosed with chlamydia to the LTK website, compared with 49% in this study [25]. This discrepancy may reflect family planning clinicians’ greater expertise in sexual health and participation in meetings and conferences where evaluations of the LTK website and other similar resources were presented. Considering most chlamydia diagnoses occur in general practice [26,27], more proactive approaches may be needed to raise awareness about the LTK website among general practitioners. Sexual health clinics are also a setting where many STI diagnoses occur, but to our knowledge there is no published information on LTK website use among clinicians in this setting. Recently an Internet-based resource and PN service for STIs called “Better to know” was also developed specifically for Aboriginal people including gender-specific information, with the option to anonymously notify partners by SMS text messaging or email [28]. Thus any promotion of such Internet-based services among clinicians should consider the options (LTK, WhyTest, Better to know) available for different target groups.

### Strengths and Limitations

The survey had a number of strengths. First, because of questionnaire administration via the clinic representative and reminder, the response rate was much higher than other postal chlamydia knowledge and practice surveys in Australian general practice settings [29-31]. Second, focus groups provided a deeper understanding of how the LTK website was integrated into routine practice and why it wasn’t. A number of limitations should also be noted. First, family planning clinicians are only a subset of Australian sexual health providers, and results may not be generalizable to other clinical settings. Second, the clinicians who did not respond to surveys or participate in focus groups may have included more part-time clinicians who had different knowledge, attitudes, and practices than those who did participate. Also, as we did not explore quantitatively whether clinicians knew about the LTK website in the survey (only if they used it), we could not formally assess if there was an association between awareness of the website and referring to it. Third, we did not collect information about state- or territory-based legislation on ability of nurses to supply treatment for people diagnosed with chlamydia. Although this could be a reason for some difference in the use of LTK between states, we do not think it would be a major one. Finally, as the study was conducted in the context of a broader study of chlamydia testing and management practices at Australian family planning clinics, only a subset of questions and a fraction of focus group interview time were dedicated to PN and the LTK website.

### Conclusions

In conclusion, the study has demonstrated that the LTK website has become an important PN tool for family planning clinicians, that it has the potential to become part of routine practice. To raise awareness of the LTK website among clinicians, the tool should be specifically mentioned in all clinic protocols and other clinical resources [29,30] and regular organizational newsletters. Also, at the bottom of the pathology reports with positive chlamydia test result, there could be a link added to the Australian STI guidelines that include information about the LTK website. The study also highlights the need for further training and education about PN generally to highlight the importance of PN for chlamydia and information (or a brief algorithm) on how best clinicians could assist their clients with PN. Further research is needed to determine the efficacy of the tool in regard to treatment of the partner and reinfection of the index case.

## Abbreviations

LTK: Let Them Know
PN: partner notification
SMS: short message service
SRH: sexual and reproductive health
STI: sexually transmitted infection
